# Case Reports and Artificial Intelligence: Picking a Pearl among Abundant Reports

**DOI:** 10.31662/jmaj.2026-0135

**Published:** 2026-05-08

**Authors:** Shigeki Matsubara

**Affiliations:** 1 Department of Obstetrics and Gynecology, Jichi Medical University, Tochigi, Japan; 2 Department of Obstetrics and Gynecology, Koga Red Cross Hospital, Koga, Japan; 3 Medical Examination Center, Ibaraki Western Medical Center, Chikusei, Japan

**Keywords:** artificial intelligence, case report, ChatGPT

## To the Editor

I welcome Mohanty’s comment ^(1)^ on my paper ^(2)^, in which I described the importance of case reports in medical publication ^(2)^. Mohanty expanded on this noting that publishing case reports can trigger vigilance for additional cases, leading to valuable original studies. I agree with this and wish to share my view. Artificial intelligence (AI) can generate case reports, which may increase the number of less significant reports, and this may threaten valuable case reports.

A physician’s gut feeling of “something different from the usual” is the origin of case reports. This arises from recognizing a deviation from textbook descriptions or from lifelong clinical experience. Case reports can be written by physicians who have been sincerely studying and working and, importantly, have closely observed “this” patient.

“Something different” must be verbalized as “known,” “unknown,” and “problem.” ^(3)^ Thus, case reports can be completed in the following order: (i) a gut feeling of something different → (ii) verbalizing it according to the paper structure ^(3)^ → (iii) an extensive literature search to confirm it → (iv) writing.

If one intentionally asks, AI could automatically perform procedures (iii) and (iv), and can even perform (ii). My previous experiment ^(4)^ showed that inputting a patient profile into ChatGPT can generate a case report, albeit at a basic level. If one inputs “known,” “unknown,” and “problem” (at stage ii), AI could write a case report of considerable quality. This suggests that if one can “feel” that there is “something different” in a certain patient, AI could write a structurally and linguistically proper case report in a short time. In extreme cases, AI can bypass the author’s sincere study and work, and close observation, which have long been prerequisites for writing case reports.

Thus, I have two concerns. First, a large number of case reports that are heavily dependent on AI may be submitted. Although experienced specialists could discern their superficial content, a considerable number of reviewers may be misled by their good structure and language. Pushed by large number of case report submissions, some journals may stop publishing them. This is a dilemma. Although AI may help some authors write, over-reliance may deprive case reports of the opportunity for publication altogether. Second, although some case reports involve “pearls” for future breakthrough studies, such pearls may be embedded within an abundance of case reports.

I do not say that AI use is wrong; however, ultimately, it is the human eye that can discern the pearls among the non-pearls.

## Article Information

### Author Contributions

Shigeki Matsubara designed this study; wrote, edited, and approved the final manuscript; and meets the ICMJE criteria for authorship.

### Conflicts of Interest

None

### Ethics Approval

Jichi Medical University does not demand IRB approval for this type of study. Patient anonymity preservation and informed consent for reporting are not applicable.

### Patient Anonymity

Not needed.

### Informed Consent

Not needed.

### Data Availability Statement

Data sharing is not applicable to this article as no new data were created or analyzed in this study.
